# Acidified biochar improves lead tolerance and enhances morphological and biochemical attributes of mint in saline soil

**DOI:** 10.1038/s41598-023-36018-2

**Published:** 2023-05-30

**Authors:** Azhar Sohail Shahzad, Uzma Younis, Nargis Naz, Subhan Danish, Asad Syed, Abdallah M. Elgorban, Rajalakshmanan Eswaramoorthy, Shoucheng Huang, Martin Leonardo Battaglia

**Affiliations:** 1 Botany Department, The Islamia University of Bahawalpur, Rahim Yar Khan, Pakistan; 2 Botany Department, The Islamia University of Bahawalpur, Bahawalpur, Pakistan; 3 Department of Soil Science, Faculty of Agricultural Sciences and Technology, Bahauddin Zakariya University, Multan, Pakistan; 4 Department of Botany and Microbiology, College of Science, King Saud University, P.O. 2455, Riyadh, 11451 Saudi Arabia; 5 Department of Biochemistry, Centre of Molecular Medicine and Diagnostics (COMMAND), Saveetha Dental College and Hospitals, Saveetha Institute of Medical and Technical Sciences (SIMATS), Chennai, 600077 India; 6 College of Life and Health Science, Anhui Science and Technology University, Fengyang, 233100 China; 7 The Nature Conservancy, Arlington, VA 22203 USA

**Keywords:** Plant sciences, Plant stress responses, Abiotic

## Abstract

Lead (Pb) toxicity is a significant environmental issue, especially in areas with a past of industrial activities and mining. The existence of Pb in the soil can have negative impacts on plant growth and development, and it can also pose a risk to human health through the food chain. Acidified carbon has shown promise as an effective management technology for mitigating Pb toxicity. This study provides important insights into the potential of acidified biochar as a low-cost and eco-friendly method for managing Pb-contaminated soils. The current study explores the effectiveness of acidified biochar (AB) in alleviating Pb stress in mint. The study involved two levels of Pb (0 = control and 200 mg/kg Pb) and four levels of AB as treatments (0, 0.45, 0.90, and 1.20%). Results indicate that 1.20% AB was the most effective treatment, significantly decreasing root and shoot Pb concentration while enhancing shoot and root fresh and dry weight, shoot and root length, and shoot and root N, P, and K concentration. Moreover, a significant decrease in MDA (0.45AB, 0.90AB, and 1.20AB caused a decline in MDA content by 14.3%, 27.8%, and 40.2%, respectively) and an increase in ascorbic acid (0.45AB, 0.90AB, and 1.20AB led to an increase in ascorbic acid content of 1.9%, 24.8%, and 28.4%, respectively) validated the effectiveness of 1.20% AB compared to the control. Adding 0.45AB, 0.90AB, and 1.20AB led to an increase in soluble sugar content of 15.6%, 27.5%, and 32.1%, respectively, compared to the treatment without AB. Further investigations at the field level are suggested to confirm the efficacy of 1.20% AB as the best treatment against Pb toxicity in saline soil conditions.

## Introduction

Peppermint (Mentha × piperita) and other members of the Lamiaceae (Labiatae) family, such as mint (*Mentha spicata*) and sage (*Salvia officinalis*), are aromatic, herbaceous plants. Mentha is derived from the Greek Mintha, a mythological nymph who was said to have transformed into a plant, and piperita is derived from the Latin piper, which signifies its aromatic and piquant taste. Mints are widely dispersed in temperate areas across Eurasia, North America, southern Africa, Asia, and Australia, with naturalization observed in numerous localities^1^. The plant is widely employed in herbal medicines, foodstuffs, beverages, teas, cosmetics, and other products^1,2^ Mentha species are the second most prolific source of essential oils globally, producing approximately 2000 tons annually. Resinous spots containing volatile oils are present in both the leaves and shoots of these species. Mentha species are the second most prolific source of essential oils globally, producing approximately 2000 tons annually. Resinous spots containing volatile oils are present in both the leaves and shoots of these species^3^. However, environmental abiotic stresses have been shown to affect its growth parameters, essential oil production, and composition^4^.

The release of numerous toxic pollutants into the atmosphere has significantly accelerated since the onset of the Industrial Revolution^5^. A heterogeneous mixture of chemical compounds and heavy metals (HMs) in the environment presents a potential hazard to human health and the environment^6,7^. The concentration of lead (Pb) in the Earth’s crust is estimated to be approximately 0.002%. While trace quantities of certain heavy metals, such as zinc (Zn) and copper (Cu), are essential components of many enzymes and biochemical processes, lead (Pb) and cadmium (Cd) are not necessary for plant metabolism. Nonetheless, high concentrations of any heavy metal can harm plant health^8^. Lead has been identified as the second most toxic metal after arsenic due to its harmful effects on life. Moreover, mining activities are a significant source of soil Pb pollution^5,9^.

On the other hand, Hhgh salinity levels can also harm soil organisms, leading to their demise and earning it the monikers of "white death" and "silent murderer"^10^. Approximately one-fifth of the world's agricultural regions and more than half of all areas subject to irrigation are affected by salinity^11^.Usually crop yields become drastically reduced due to salinization or heavy metal accumulation, rendering crop production economically unsustainable^12,13^. If lead toxicity is accompanied by salinity, it can significantly affect morphological and physiological reactions, including alterations in growth, water availability and root cell uptake, photosynthetic efficiency, carbon allocation, and utilization^14–17^. Plants may exhibit aberrant behavior in various processes, including signal transduction, gene expression, reactive oxygen species accumulation, metabolic and physiological pathways, lipid membrane oxidation, wilting, and mortality^10^. The ability of different species to cope with metal and salt stress is contingent upon their genotype and variety^10,16^. At below or moderate level concentrations of salinity and heavy metals (Pb), no visible evidence exists of stress-induced damage to plant leaves^18^.

Most researchers advocated the incorporation of acidified biochar as a soil amendment to mitigate lead toxicity in alkaline soils^19,20^. Biochar, a unique multifunctional carbon compound, has been extensively utilized as a soil amendment to enhance soil quality due to its remarkable characteristics^21,22^. Incorporating acidified biochar into soil effectively decreases fertilizer application and volatilization losses^22^. The application of acidified biochar has been demonstrated to alter soil's physical, chemical, and biological characteristics, including consistency, composition, pore size distribution, density, nutrient status, and carbon sequestration^23–25^. Applying biochar to soils significantly decreased the soil pH and plant-available Fe concentrations while increasing the availability of P, K, Mg, Ca, Zn, Cu, and Mn to plants. The present study addressed the knowledge gap concerning the effects of acidified biochar on various growth parameters of mint. This experiment aimed to evaluate and determine the optimal application rate of acidified biochar to enhance the morphological, physiological, and biochemical characteristics of mint under saline and Pb contaminated soil conditions of the IUB RYK campus. It is hypothesized that using acidified biochar could increase mint's morphological, physiological, and biochemical characteristics under lead contaiminated and saline soil conditions of the IUB RYK campus.

## Materials and methodology

### Biochar production and characterization

The pyrolysis of crumpled cotton sticks, collected following the crop's fruitage during February 2022, was conducted in a specially designed stainless-steel furnace with restricted air feed. The process was completed at 723 K for two hours. The resulting char was then acidified by submerging it in H_2_SO_4_ for 12 h before oven-drying it for 48 h at 65 °C. After cooling, the char was minced to 2 mm and stored for future use. As outlined by McLaughlin et al.^26^, gravimetric analysis was utilized to determine the various components of biochar. Aliquots of a 1:10 mixture of biochar and distilled water were used to ascertain the pH^27^ and EC^28^ of biochar.

Additionally, elemental analysis was employed to measure the total carbon concentration of biochar. The Biochar model is digested and then distilled using the Kjeldahl distillation by Schouwvenberg^29^ method to determine the N concentration in the biochar. The phosphorus (P) and potassium (K^+^) concentration in biochar was determined using HNO_3_–HClO_4_^30^. Subsequently, a spectrophotometer was used to analyze the phosphorus (P) absorbance employing the Ammonium Vanadate-Ammonium Molybdate yellow color technique, whereas the potassium (K^+^) concentration was determined with a flame photometer^31^. The physiochemical characteristics of biochar are provided in Table 1.

**Table 1:** Pre-experimental soil, biochar, and irrigation characteristics.

Soil	Values	Biochar	Values	Irrigation	Values
pH	8.60	pH	6.05	pH	7.65
EC*e* (dS/m)	1.88	EC*e* (dS/m)	3.55	EC (µS/cm)	465
SOC (%)	0.21	Ash content (%)	33	Carbonates (meq./L)	0.00
TN (%)	0.02	Volatile matter (%)	21	Bicarbonates (meq./L)	5.12
EP (µg/g)	10.12	Fixed carbon (%)	46	Chloride (meq./L)	0.15
AK (µg/g)	92	TN (%)	2.15	Ca + Mg (meq./L)	4.10
Sand (%)	50	TP (%)	1.05	Sodium (mg/L)	363
Silt (%)	20	TK (%)	4.41		
Clay (%)	30	Surface area (m^2^/g)	350		
Texture	Sandy clay loam	CEC (meq./100 g)	400		

*TN* total nitrogen, *EP* extractable phosphorus, *AK* available potassium, *CEC* cation exchange capacity, *EC* electrical conductivity.

### Fourier transform infrared spectroscopy (FTIR) for biochar

The functional groups present in biochars were observed using Fourier transform infrared spectroscopy (FTIR)^32^. To prepare the samples for analysis, 1.5–2.0 g of each sample was mixed with 200 mg of potassium bromide (KBr) and ground finely. The resulting mixture was compressed under vacuum using a standard device with a pressure of 75 kN cm^−2^ for 2–3 min to create pellets for spectral analysis. The spectra were obtained using a scanning range of 400–4000 cm^−1^ with a resolution of 4 cm^−1^.

### Collection of soil for research experiment

Soil samples were collected^33^ to a depth of 0–15 cm from the vicinity of the departmental building adjacent to the nursery area of the IUB RYK Campus. The samples were then sieved through a 5 mm sieve and artificially contaminated with metal salts during the soil preparation for pots.

### Plant materials

Rhizomes of *Mentha piperita* L. (mint) were purchased from a nursery in Sadiqabad (28°0.2814 N, 69°0.5910 E) to be employed in this experimental investigation.

#### Experimental design and pot preparation

A controlled experiment was conducted at the Botanical Garden of The IUBRYK Campus to evaluate the effects of acidified biochar (0, 0.45, 0.90, and 1.20% w/w) and lead (200 mg Pb/kg soil using Pb (NO_2_)_3_ (CAS Number:10099-74-8; Batch Number: BCCJ5381; 3050 Spruce Street, Saint Louis, MO 63103, USA) on *Mentha piperita* L. (mint) growth. Five kilogram of artificial metal-contaminated soil (prepared with lead nitrate), was combined with the appropriate biochar treatment in four replicate clay pots as per treatment plan. The soil water holding capacity (WHC) was maintained at 60% through frequent irrigation. Healthy *Mentha piperita* L. rhizomes were divided into 2–3 inches lengths and propagated in clay pots.

#### Growth experiment

On 21-6-2022, commonplace practices planted five *Mentha piperita* L. rhizomes into the ground to a depth of two inches. Ten days after the sprouts had emerged, the rhizome count per container was reduced from seven to five. To mitigate the effects of the local temperature, the containers were situated outdoors in a shaded area. To sustain each pot’s WHC (water-holding capacity), they were rinsed periodically to maintain a humidity content of 60%.

#### Harvesting

Upon reaching maturity (57 days post-sowing), the mint plants were harvested on 16 August 2022. Subsequently, the fresh biomasses of mint roots and shoots were weighed using an electrical balance. Later, the plant samples were dried at 65 °C for 24 h to obtain the dry biomasses of mint shoots and roots.

#### Analysis of pigments in mint

An aliquot of 0.2 g leaf sample was homogenized using a pestle and mortar and extracted overnight with 4 mL of 80% acetone. The homogenized solution was then filtered, and the filtrate was supplemented with 80% acetone to bring the volume to 20 mL. The absorption of the chlorophyll extract was measured using a Perkin-Elmer Optima 2100 DV spectrophotometer at wavelengths 663, 645, and 480 nm. The chlorophyll content (a, b, and total) was calculated using the corresponding equations^34^.

$${\text{Chlorophyll}} \rm{''}a{''}\left( {{\text{mg g}}^{ - 1} {\text{F}}.{\text{W}}} \right) = \left[ {1.27\left( {{\text{OD}}663} \right) - 2.69\left( {{\text{OD}}645} \right)} \right] \times {\text{V}}/1000 \times {\text{W}}$$

$${\text{Chlorophyll}} \rm{''}b{''}\left( {{\text{mg g}}^{ - 1} {\text{ F}}.{\text{W}}} \right) = \left[ {22.9\left( {{\text{OD}}645} \right) - 4.68\left( {{\text{OD}}663} \right)} \right] \times {\text{V}}/1000 \times {\text{W}}$$

$${\text{Total chlorophyll}}\left( {{\text{mg g}}^{ - 1} {\text{ F}}.{\text{W}}} \right) = \left[ {20.2\left( {{\text{OD }}645} \right) + 8.02\left( {{\text{OD }}663} \right)} \right] \times {\text{V}}/1000 \times {\text{W}}$$

$${\text{Carotenoids }}\left( {{\text{mg g}}^{ - 1} {\text{ F}}.{\text{W}}} \right){ } = {\text{ OD }}\left( {480} \right){ } + { }\left( {0.114{ } \times {\text{ OD }}663} \right){ } - { }\left( {0.638{ } \times {\text{ OD }}645} \right){\text{ V }}/{ }\left( {1000{ } \times {\text{ W}}} \right)$$;$$ V = Filrate\;volume\left( {{\text{ml}}} \right)$$ $$W = Leaf\;weight\left( {\text{g}} \right)$$; $$OD = Optical\;densit$$.

#### Ascorbic acid 

The ascorbic acid content in 5 g of fresh leaves was quantified by homogenizing them in an extracting mixture (5 g of oxalic acid, 0.75 g of the sodium salt of EDTA in 100 mL of distilled water) and subsequently centrifuging for 15 min at 682.2 rads^−1^. One mL of homogenate was then mixed with 5 mL of DCPIP (Dichlorophenol indophenol), which resulted in the homogenate becoming pink in color, and the absorbance of the solution was measured at 520 nm. To render the homogenate colorless, 1% (C_6_H_8_O_6_) ascorbic acid solution was added, and the absorbance of the colorless solution was recorded at the same wavelength. A standard curve was then plotted utilizing solutions of varying dilutions of ascorbic acid (0.01–0.07 g L^−1^), and the ascorbic acid content was calculated following the method of Keller and Schwager^35^.

$${\text{Ascorbic}}\;{\text{acid }}\left( {{\text{mg g}}^{ - 1} {\text{ F}}.{\text{W}}} \right) = \left[ {\left( {\left( {{\text{Eo}} - {\text{Es}} - {\text{Et}}} \right){\text{V}}/100 \times {\text{W}}} \right) \times 100} \right]$$

#### Total proteins

A homogenate of 0.2 g of fresh leaves and 4 mL of phosphate buffer was prepared in a pestle and mortar and centrifuged at 6000 rpm for 10 min. The Bradford formula (1976) was employed to calculate the total soluble protein content in the supernatant, and the absorbance was measured at 595 nm with a UV-1900 BMS spectrophotometer.

$${\text{Total}}\;{\text{soluble}}\;{\text{protein }}\left( {{\text{mg g}}^{ - 1} {\text{ F}}.{\text{W}}} \right) = {\text{Sample}}\;{\text{reading}} \times {\text{Sample}}\;{\text{vol}}. \times \frac{{{\text{Dilution}}\;{\text{factor}}}}{{\text{W}}} \times 1000$$

$${\text{Total}}\;{\text{amino}}\;{\text{acids}} = {\text{sample}}\;{\text{reading}} \times {\text{sample}}\;{\text{volume}} \times {\text{dilution}}\;{\text{factor}}/{\text{W}} \times 100$$

#### Malondialdehyde (MDA)

The amount of malondialdehyde (MDA) in leaf tissue was quantified to assess the degree of lipid peroxidation of the membrane attributed to salinity-induced reactive oxygen species (ROS) damage, following the protocol outlined by Cakmak and Horst^37^. This involved homogenizing 1 g of leaf tissue in 3 ml of 0.1% trichloroacetic acid (TCA) solution. The homogenate was centrifuged for 15 min at a relative centrifugal force of 20,000 × g. Subsequently, 0.5 mL of the resulting supernatant was mixed with 3 mL of a 0.5% Thiobarbituric acid solution containing 20% TCA. The mixture was incubated for 50 min in a shaking water bath at 95 °C. The tubes were immediately cooled in an ice bath before being centrifuged for 10 min at a relative centrifugal force of 10,000 × g. The absorbance of the sample was then measured at 532 and 600 nm. The malondialdehyde (MDA) content was subsequently calculated using the following formula.

$${\text{MDA}}\,{\text{level }}\left( {{\text{nmol}}} \right) = \Delta \left( {{\text{A}}532\,{\text{nm}} - {\text{A}}600\;{\text{nm}}} \right)/1.56 \times 10.5$$

The absorption coefficient for measuring MDA is 156 mmol^−1^ cm^−1^.

#### Soluble sugars

Using 80% acetone obtained a mass of 0.1 g of leaf extract. The sample was heated at 600 °C for 6 h. Subsequently, 6 ml of anthrone reagent was amalgamated with 1 ml leaf extract in a glass tube. The tube was then boiled in boiling water for 10 min. Afterward, the test tubes were kept at room temperature for 20 min, then instant cooling in an ice bath for 10 min. The absorbance of the solution was recorded at 625 nm using a spectrophotometer. The approach above generated the standard curve, and the soluble sugar content was calculated using the arch.

### Determination of total nitrogen in mint leaves

The nitrogen content of mint plants was quantitatively determined using Kjeldhal's distillation apparatus following Bremner’s^38^ methodology.

### Digestion mixture preparation

To achieve the desired goal, a digestion mixture was prepared with 0.42 g of Se, 14 g of LiSO_4_.2H_2_O, and 0.42 g of selenium, to which 350 mL of H_2_O_2_ was added. A precise volume of 420 mL of concentrated sulfuric acid was added to the mixture and stirred in an ice bath to mitigate the exothermic reaction. The mixture was then stored in a refrigerator for subsequent analysis.

### Digestion

Approximately 0.2 g of dry sample material and 1 mL of digestion mixture were added to the digestion flasks for the roots and shoots. Subsequently, the digesting flasks were placed on the hot plate and heated to a temperature of 200 °C. Upon the plant material turning black, 0.5 mL of HClO_4_ was added to the digestion flask. Once the plant material had become colorless, the digesting flasks were removed from the hot plate, and the requisite volume was established for further analysis.

### Distillation

About 5 mL of the digested sample and 5 mL of a 5N NaOH solution were added to a Kjeldahl flask to distill the samples. A bromothymol blue solution and a 4% boric acid solution were inserted into the collecting tube. After collecting 35–40 mL of distillate, the answer was titrated with 0.01N H_2_SO_4_ solution. Subsequently, the mg kg^−1^ Nitrogen content in the plant samples was determined using the corresponding equation.

$${\text{N }}\left( {\text{\% }} \right)  = \frac{{\left( {{\text{a}} - {\text{c}}} \right) \times {\text{N}} \times {\text{Total}}\;{\text{vol}}.{ } \times 14000}}{{{\text{aliquot}} \times {\text{sample}}\;{\text{weight }}\left( {\text{g}} \right)}} $$

a = mL of acid for sample; N = Normality of acid; c = mL of acid for blank.

### Determination of total phosphorus in mint leaves

A spectrophotometer was calibrated using KH_2_PO_4_ standards of concentrations 2, 4, 6, 8, 10, and 12 ppm. Barton's reagent was then added to 1 mL of ordinary KH_2_PO_4_ solutions and previously digested plant shoots and roots, and the total volume was decreased to 10 mL using deionized water. The readings from the samples were then taken after calibrating the apparatus at 470 nm wavelength, and a reference curve was generated for reading correction. To calculate the mg kg^−1^ P, the following equation was used:

$${\text{P }}\left( {{\text{mg}}\;{\text{kg}}^{ - 1} } \right) = \frac{{\left( {{\text{Absorbance}}} \right){\text{ reading }} \times {\text{dilution }}\;{\text{factor}}}}{{{\text{Sample}}\;{\text{weight}}\;{\text{initially}}\;{\text{taken}}\left( {\text{g}} \right)}}$$

#### Determination of total potassium in mint leaves

The flame photometer was initially calibrated using KCl standard solutions with concentrations of 0, 2, 4, 6, 8, and 10 ppm, following the procedure outlined by Jones et al.^39^. Subsequently, the digested plant material was diluted according to the specifications, and the potassium levels were measured using the flame photometer. Finally, the percentage K was calculated using the established formula.

$$ {\text{K }}\left( {\text{\% }} \right) = {\text{K ppm }} \times {\text{dilution factor}} $$

#### Pb analysis of mint

The plant samples were initially air-dried and then split into 0.5 g for digestion. Subsequently, the models were oven-dried for 24 h at 65 °C, and a di-acid solution (HNO_3_:HClO_4_) was added at 10 ml per minute. The samples were left overnight to facilitate the softening of the plant tissues. Following this, the samples were placed on a heated plate at 50 °C, which was progressively raised to 280 °C after 24 h. When the plant samples began to soften, and white odors were emitted, they were removed and cooled. After cooling, the necessary volume was obtained for further AAS analysis^40^. To determine the metal uptake by mint, the nutrient concentrations (g/g DW) in the shoot or root were multiplied by the biomass of the respective organs^41^.

### Statistical analysis

Standard statistical analyses were done for the collected data^42^. The study employed OriginPro statistical software program^43^ to analyze variance using a two-way ANOVA and a Fisher LSD test; *p* ≤ 0.05 to identify any statistically significant differences between the treatments.

### Ethics approval and consent to participate

We all declare that manuscript reporting studies do not involve any human participants, human data, or human tissue. So, it is not applicable.

### Experimental research and field studies on plants (either cultivated or wild), including the collection of plant material, must comply with relevant institutional, national, and international guidelines and legislation

Experimental research and field studies on plants including the collection of plant material are comply with relevant institutional, national, and international guidelines and legislation.

## Results

### Shoot fresh weight

**Figure 1:**
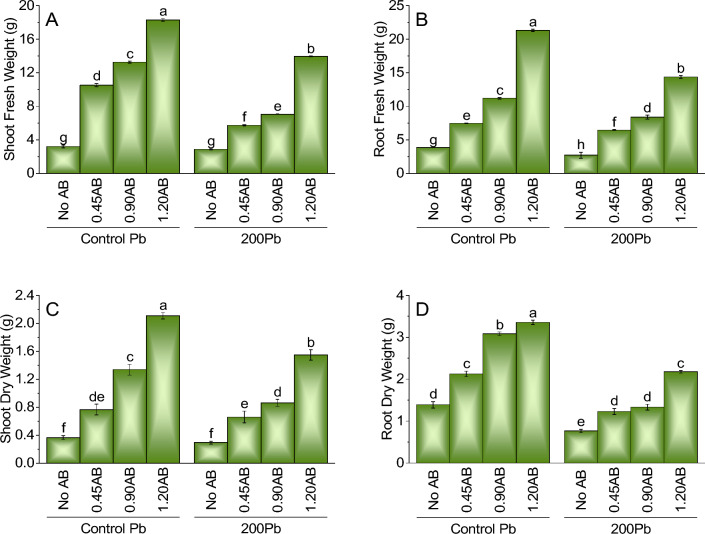
The impact of various levels of acidified biochar (0%, 0.45%, 0.90%, 1.20%) on the growth of mint plants in both saline and lead-contaminated saline soil. The plant growth parameters assessed were the fresh weight of shoots (**A**), roots (**B**), dry weight of shoots (**C**) and roots (**D**).To determine the significance of the differences between the various acidified biochar application levels, Fisher’s LSD analysis was employed, and the bars on the graphs show the letters that correspond to statistically significant differences (*p* ≤ 0.05).

The experiment results show that the addition of acidified biochar had a significant effect on both the lead content and shoot fresh weight of the plants. When comparing the control treatment of Pb without AB to the addition of 0.45AB, 0.90AB, and 1.20AB, there were increases in lead content by 226.06%, 312.24%, and 467.76%, respectively. Similarly, there were corresponding increases in shoot fresh weight of 226.06%, 312.24%, and 467.76%, respectively. The negative effect of lead on shoot fresh weight was mitigated when acidified biochar was added to the system. When 200Pb was added to the system without AB, there was a significant decrease in shoot fresh weight by 89.77% compared to the control treatment of Pb without AB. However, adding 0.45AB, 0.90AB, and 1.20AB led to increases in shoot fresh weight of 77.98%, 118.58%, and 333.29%, respectively (Fig. 1A).

### Root fresh weight

A comparison between the control treatment of Pb without AB and the addition of 0.45AB, 0.90AB, and 1.20AB revealed significant increases in lead content by 94.57%, 190.09%, and 453.55%, respectively. Corresponding increases in root fresh weight were also observed, with percentages of 40.22%, 44.65%, and 83.47%, respectively. Adding acidified biochar to the system appeared to mitigate the negative effect of lead on root fresh weight. When 200Pb was added to the system without AB, a significant decrease in root fresh weight of 29.59% was observed compared to the control treatment of Pb without AB. However, adding 0.45AB, 0.90AB, and 1.20AB led to increased root fresh weight of 123.31%, 191.39%, and 148.96%, respectively (Fig. 1B).

### Shoot dry weight

Adding acidified biochar (AB) significantly increased shoot dry weight compared to the control treatment of Pb without AB. In particular, adding 0.45AB, 0.90AB, and 1.20AB led to increases in shoot dry weight of 108.49%, 264.20%, and 472.09%, respectively, compared to the control Pb treatment without AB. Similarly, adding AB also mitigated the negative effect of lead on shoot dry weight. When 200Pb was added to the system without AB, there was a significant decrease in shoot dry weight by 20.28% compared to the control treatment of Pb without AB. However, adding 0.45AB, 0.90AB, and 1.20AB led to increased shoot dry weight of 125.85%, 194.91%, and 320.43%, respectively (Fig. 1C).

### Root dry weight

The results suggest that the addition of acidified biochar (AB) can effectively mitigate the negative effects of lead (Pb) contamination on root dry weight. The addition of 0.45AB, 0.90AB, and 1.20AB to the Pb contaminated soil led to increases in root dry weight of 52.38%, 121.43%, and 152.38%, respectively, compared to the control treatment of Pb without AB. Similarly, when 200Pb was added to the system without AB, there was a decrease in root dry weight by 44.67% compared to the control treatment of Pb without AB. However, the addition of 0.45AB, 0.90AB, and 1.20AB led to increases in root dry weight of 59.74%, 72.38%, and 57.14%, respectively, indicating the positive effect of AB on root dry weight (Fig. 1D).

### Chlorophyll a

**Figure 2:**
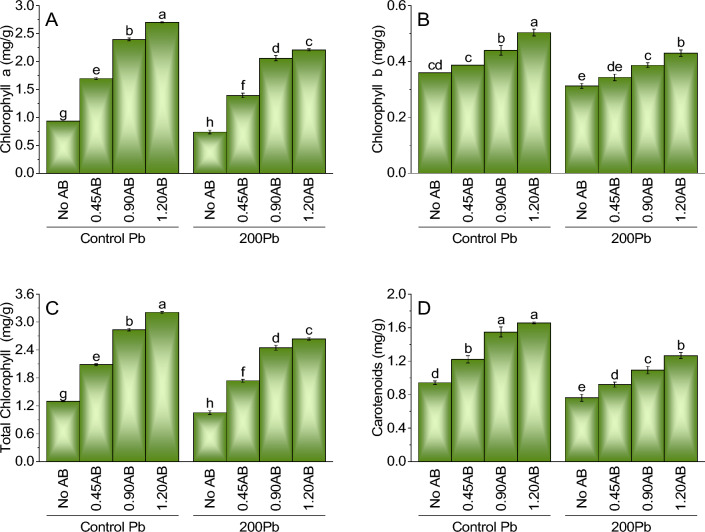
The impact of various levels of acidified biochar (0%, 0.45%, 0.90%, 1.20%) on the growth of mint plants in both saline and lead-contaminated saline soil. The plant growth parameters assessed were the chlorophyll a (**A**),chlorophyll b (**B**), total chlorophyll (**C**) and carotenoids (**D**). To determine the significance of the differences between the various acidified biochar application levels, Fisher’s LSD analysis was employed, and the bars on the graphs show the letters that correspond to statistically significant differences (*p* ≤ 0.05).

The addition of acidified biochar to lead-contaminated soil positively affected chlorophyll a content in plants. Compared to the control, adding 0.45AB, 0.90AB, and 1.20AB led to significant increases in chlorophyll a content of 81.6%, 156.2%, and 189.1%, respectively. Additionally, when 200Pb was added to the system without AB, there was a significant decrease in chlorophyll a content by 20.9% compared to the control treatment of Pb without AB. However, the addition of 0.45AB, 0.90AB, and 1.20AB led to increases in chlorophyll a content of 88.2%, 178.4%, and 157.4%, respectively. These findings suggest that the addition of acidified biochar can effectively mitigate the negative impacts of lead on chlorophyll a content in plant (Fig. 2A).

### Chlorophyll b

For chlorophyll b, the results showed that the addition of acidified biochar (AB) to the soil contaminated with lead (Pb) had a limited effect. Compared to the control treatment of Pb without AB, there were only slight increases in chlorophyll b content with the addition of 0.45AB, 0.90AB, and 1.20AB. The percentage increases were 7.2%, 22.5%, and 40.1%, respectively. Similarly, when 200Pb was added to the system without AB, there was a decrease in chlorophyll b content by 9.9% compared to the control treatment of Pb without AB. However, the addition of 0.45AB, 0.90AB, and 1.20AB led to slight increases in chlorophyll b content of 9.7%, 22.8%, and 19.9%, respectively. Overall, the results suggest that the addition of acidified biochar had a limited effect on chlorophyll b content in plants under lead stress (Fig. 2B).

### Total chlorophyll

Compared to the control treatment of Pb without AB, adding acidified biochar (AB) to lead (Pb) contaminated soil increased total chlorophyll content. Specifically, the addition of 0.45AB, 0.90AB, and 1.20AB led to increases in total chlorophyll content of 60.7%, 118.8%, and 147.4%, respectively. In contrast, when 200Pb was added to the system without AB, there was a decrease in total chlorophyll content by 18.7% compared to the control treatment of Pb without AB. However, the addition of 0.45AB, 0.90AB, and 1.20AB led to increases in total chlorophyll content of 65.6%, 88.9%, and 104.7%, respectively (Fig. 2C).

### Carotenoids

Introducing acidified biochar (AB) into lead-contaminated and non-contaminated soil led to an increase in carotenoid content compared to the control treatment of Pb without AB. Specifically, adding 0.45AB, 0.90AB, and 1.20AB resulted in carotenoid content increases of 29%, 64%, and 76%, respectively. On the other hand, introducing 200Pb into the system without AB resulted in a 19.1% reduction in carotenoid content compared to the control treatment of Pb without AB. However, adding 0.45AB, 0.90AB, and 1.20AB resulted in carotenoid content increases of 20.6%, 16.1%, and 34.3%, respectively (Fig. 2D).

### Soluble sugar content

**Figure 3:**
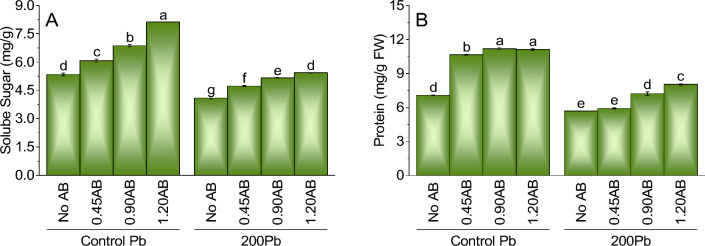
The impact of various levels of acidified biochar (0%, 0.45%, 0.90%, 1.20%) on the growth of mint plants in both saline and lead-contaminated saline soil. The plant growth parameters assessed were the soluble sugar (**A**), and protein (**B**). To determine the significance of the differences between the various acidified biochar application levels, Fisher’s LSD analysis was employed, and the bars on the graphs show the letters that correspond to statistically significant differences (*p* ≤ 0.05).

In the control Pb treatment, the addition of 0.45AB, 0.90AB, and 1.20AB led to an increase in soluble sugar content by 13.6%, 28.5%, and 51.9%, respectively, compared to the treatment without AB. The highest soluble sugar content was observed in the treatment with 1.20AB, which had a significantly higher content than all other treatments. In the 200Pb treatment, there was a decrease in soluble sugar content by 23.3% when no AB was added compared to the control treatment of Pb without AB. However, the addition of 0.45AB, 0.90AB, and 1.20AB led to an increase in soluble sugar content of 15.6%, 27.5%, and 32.1%, respectively, compared to the treatment without AB. The highest soluble sugar content was observed in the treatment with 1.20AB, which had a significantly higher content than all other treatments except for 0.90AB (Fig. 3A).

### Protein content

The protein content data shows that adding acidified biochar (AB) to lead-contaminated soil positively affects protein synthesis in plants. Compared to the control treatment of Pb without AB, adding 0.45AB, 0.90AB, and 1.20AB led to increases in protein content of 50.1%, 57.6%, and 57.0%, respectively. Similarly, when 200Pb was added to the system without AB, there was a decrease in protein content by 19.2% compared to the control treatment of Pb without AB. However, the addition of 0.45AB, 0.90AB, and 1.20AB led to increases in protein content of 4.1%, 27.6%, and 40.2%, respectively. These results suggest that the addition of acidified biochar can mitigate the negative effects of lead on protein synthesis in plants. The highest protein contents were observed in the treatments with 0.90AB and 1.20AB, indicating that these levels of biochar addition may be optimal for enhancing protein synthesis in plants (Fig. 3B).

### Malondialdehyde

**Figure 4:**
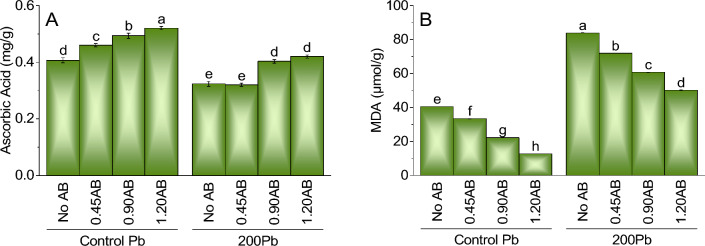
The impact of various levels of acidified biochar (0%, 0.45%, 0.90%, 1.20%) on the growth of mint plants in both saline and lead-contaminated saline soil. The plant growth parameters assessed were the ascorbic acid (**A**) and MDA (**B**). To determine the significance of the differences between the various acidified biochar application levels, Fisher’s LSD analysis was employed, and the bars on the graphs show the letters that correspond to statistically significant differences (*p* ≤ 0.05).

The findings for the content of malondialdehyde (MDA) indicate that the use of acidified biochar (AB) has a considerable impact on reducing MDA levels in soil contaminated with lead. Compared to the control treatment of Pb without AB, adding 0.45AB, 0.90AB, and 1.20AB resulted in a significant decrease in MDA content by 17.6%, 45.1%, and 68.4%, respectively. Likewise, the addition of 200Pb to the system without AB led to a considerable rise in MDA content by 106.3% compared to the control treatment of Pb without AB. Nevertheless, the application of 0.45AB, 0.90AB, and 1.20AB caused a decline in MDA content by 14.3%, 27.8%, and 40.2%, respectively, indicating that acidified biochar has the potential to alleviate the adverse effects of lead on the oxidative stress in plants (Fig. 4A).

### Ascorbic acid

For ascorbic acid content, the addition of acidified biochar (AB) significantly affected the levels of ascorbic acid. Compared to the control treatment of Pb without AB, adding 0.45AB, 0.90AB, and 1.20AB led to an increase in ascorbic acid content of 13.0%, 21.2%, and 26.7%, respectively. Similarly, when 200Pb was added to the system without AB, there was a decrease in ascorbic acid content of 20.6% compared to the control treatment of Pb without AB. However, the addition of 0.45AB, 0.90AB, and 1.20AB led to an increase in ascorbic acid content of 1.9%, 24.8%, and 28.4%, respectively. These findings suggest that the addition of acidified biochar can help enhance the production of ascorbic acid in plants under lead stress (Fig. 4B).

### Shoot N

**Figure 5:**
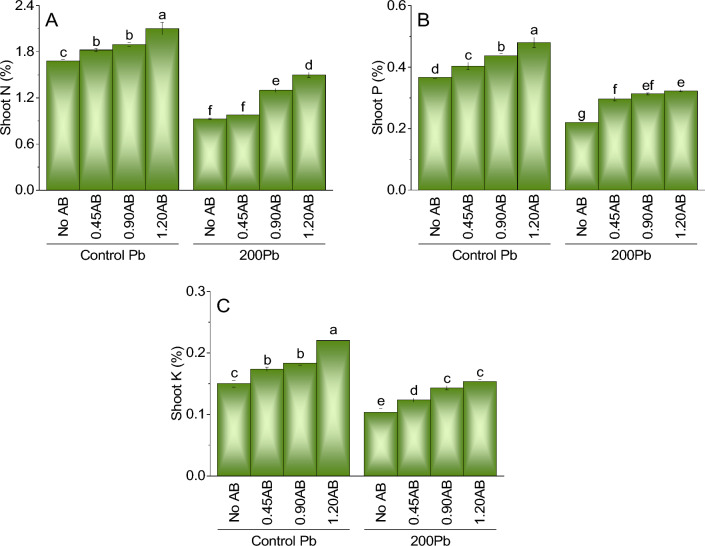
The impact of various levels of acidified biochar (0%, 0.45%, 0.90%, 1.20%) on the growth of mint plants in both saline and lead-contaminated saline soil. The plant growth parameters assessed were the shoot N (**A**) shoot P (**B**) and shoot K (**C**). To determine the significance of the differences between the various acidified biochar application levels, Fisher’s LSD analysis was employed, and the bars on the graphs show the letters that correspond to statistically significant differences (*p* ≤ 0.05).

In comparison to the control treatment of Pb without AB, the inclusion of 0.45AB, 0.90AB, and 1.20AB resulted in a marginal rise in shoot N concentration of 8.8%, 12.4%, and 25.4%, respectively. Additionally, when 200Pb was introduced to the system without AB, there was a significant reduction in shoot N concentration by 44.9% as compared to the control treatment of Pb without AB. However, the addition of 0.45AB, 0.90AB, and 1.20AB led to an upsurge in shoot N concentration of 5.8%, 39.8%, and 53.4%, respectively, which implies that acidified biochar can serve as a solution for combating the adverse effects of lead on plant growth and N uptake (Fig. 5A).

### Shoot P

The results for shoot phosphorus (P) concentration suggest that adding acidified biochar (AB) may positively impact the growth of plants under lead stress. Compared to the control treatment of Pb without AB, adding 0.45AB, 0.90AB, and 1.20AB led to a slight increase in shoot P concentration of 10.1%, 19.0%, and 31.7%, respectively. Similarly, when 200Pb was added to the system without AB, there was a significant decrease in shoot P concentration by 40.2% compared to the control treatment of Pb without AB. However, the addition of 0.45AB, 0.90AB, and 1.20AB led to an increase in shoot P concentration of 35.4%, 42.8%, and 46.0%, respectively, indicating that acidified biochar can help mitigate the negative effects of lead on plant growth and P uptake (Fig. 5B).

### Shoot K

The findings regarding shoot potassium (K) concentration imply that the use of acidified biochar (AB) could be beneficial for enhancing plant growth under lead stress. Compared to the control treatment of Pb without AB, the addition of 0.45AB, 0.90AB, and 1.20AB resulted in a slight rise in shoot K concentration of 15.5%, 22.2%, and 46.7%, respectively. Likewise, adding 200Pb to the system without AB led to a significant decline in shoot K concentration by 31.1% compared to the control treatment of Pb without AB. Nonetheless, incorporating 0.45AB, 0.90AB, and 1.20AB to the system led to an increase in shoot K concentration by 19.9%, 32.2%, and 39.6%, respectively, signifying that the use of acidified biochar could potentially alleviate the adverse effects of lead on plant growth and K uptake (Fig. 5C).

### Root N

**Figure 6:**
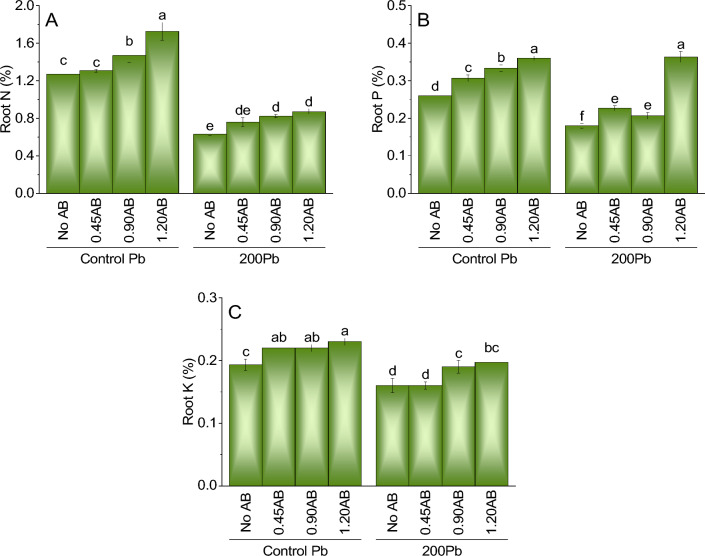
The impact of various levels of acidified biochar (0%, 0.45%, 0.90%, 1.20%) on the growth of mint plants in both saline and lead-contaminated saline soil. The plant growth parameters assessed were the root N (**A**) shoot P (**B**) and shoot K (**C**). To determine the significance of the differences between the various acidified biochar application levels, Fisher’s LSD analysis was employed, and the bars on the graphs show the letters that correspond to statistically significant differences (*p* ≤ 0.05).

For root nitrogen (N) concentration the addition of acidified biochar (AB) induced positive influence. Compared to the control treatment of Pb without AB, the addition of 0.45AB, 0.90AB, and 1.20AB resulted in a slight increase in root N concentration of 2.6%, 15.4%, and 35.4%, respectively. Likewise, the addition of 200Pb without AB led to a significant decline in root N concentration by 50.4% compared to the control treatment of Pb without AB. Nonetheless, the addition of 0.45AB, 0.90AB, and 1.20AB led to an increase in root N concentration of 20.6%, 29.3%, and 36.3%, respectively. These findings suggest that acidified biochar can alleviate the harmful effects of lead on plant growth and N uptake in roots (Fig. 6A).

### Root P

Compared to the control treatment of Pb without AB, the addition of 0.45AB, 0.90AB, and 1.20AB led to significant increases in shoot N concentration by 8.8%, 12.4%, and 25.4%, respectively. Additionally, the addition of AB significantly increased shoot K concentration by 15.5%, 22.2%, and 46.7% for 0.45AB, 0.90AB, and 1.20AB treatments, respectively. Similar trends were observed for shoot P concentration with increases of 17.4%, 27.3%, and 43.5% for 0.45AB, 0.90AB, and 1.20AB treatments, respectively.Moreover, the addition of AB led to increases in root N concentration by 2.6%, 15.4%, and 35.4% for 0.45AB, 0.90AB, and 1.20AB treatments, respectively, compared to the control treatment of Pb without AB. Similarly, the addition of AB resulted in significant increases in root K concentration by 5.8%, 15.4%, and 35.4% for 0.45AB, 0.90AB, and 1.20AB treatments, respectively. For root P concentration, the addition of AB resulted in significant increases of 17.4%, 27.3%, and 43.5% for 0.45AB, 0.90AB, and 1.20AB treatments, respectively (Fig. 6B).

### Root K

The results for root potassium (K) concentration suggest that the addition of acidified biochar (AB) did not significantly impact the growth of plants under lead stress. Compared to the control treatment of Pb without AB, adding 0.45AB, 0.90AB, and 1.20AB did not result in a significant increase in root K concentration, with only a slight increase of 14.3%, 14.3%, and 19%, respectively. In the absence of AB, the addition of 200Pb to the system resulted in a significant decrease in root K concentration by 17.1% compared to the control treatment of Pb without AB. However, the addition of AB did not significantly improve root K uptake, with only a slight increase of 0%, 18.8%, and 1.1% for 0.45AB, 0.90AB, and 1.20AB, respectively. These findings indicate that acidified biochar may not significantly affect the mitigation of lead-induced stress on plant growth and K uptake in roots (Fig. 6C).

### Shoot Pb

**Figure 7:**
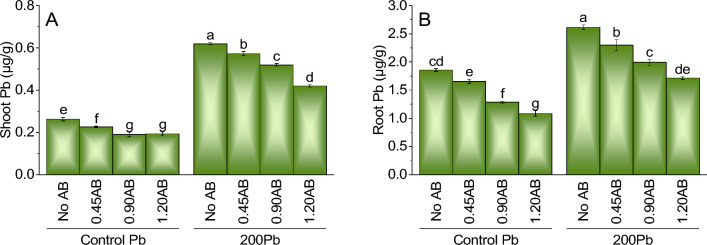
The impact of various levels of acidified biochar (0%, 0.45%, 0.90%, 1.20%) on the growth of mint plants in both saline and lead-contaminated saline soil. The plant growth parameters assessed were the shoot Pb (**A**) and root Pb (**B**). To determine the significance of the differences between the various acidified biochar application levels, Fisher’s LSD analysis was employed, and the bars on the graphs show the letters that correspond to statistically significant differences (*p* ≤ 0.05).

When compared to the control treatment of Pb without AB, the addition of 0.45AB, 0.90AB, and 1.20AB led to a slight decrease in shoot Pb concentration of 14.1%, 27.9%, and 26.6%, respectively, suggesting that acidified biochar may have limited ability to mitigate the negative effects of lead on Pb uptake in shoots. However, when 200Pb was added to the system without AB, there was a significant increase in shoot Pb concentration by 136.1% compared to the control treatment of Pb without AB. The addition of 0.45AB, 0.90AB, and 1.20AB led to a decrease in shoot Pb concentration of 8.4%, 18.1%, and 26.9%, respectively, indicating that acidified biochar may help mitigate the positive effects of lead on Pb uptake in shoots (Fig. 7A).

### Root Pb

The results for root lead (Pb) concentration suggest that adding acidified biochar (AB) may potentially reduce Pb uptake in plants under lead stress. Compared to the control treatment of Pb without AB, adding 0.45AB, 0.90AB, and 1.20AB led to a slight decrease in root Pb concentration of 10.8%, 30.5%, and 41.2%, respectively. Similarly, when 200Pb was added to the system without AB, there was a significant increase in root Pb concentration by 41.2% compared to the control treatment of Pb without AB. However, the addition of 0.45AB, 0.90AB, and 1.20AB led to a decrease in root Pb concentration of 12.3%, 31.4%, and 41.6%, respectively, indicating that acidified biochar can potentially mitigate the negative effects of lead on Pb uptake in roots (Fig. 7B).

**Figure 8:**
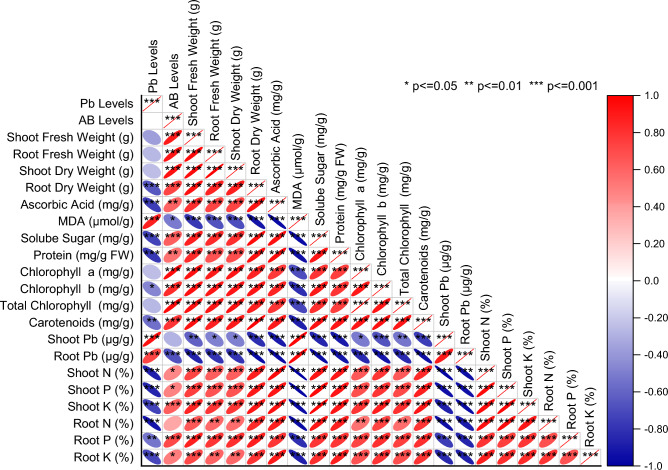
Pearson correlation for various levels of acidified biochar (0%, 0.45%, 0.90%, 1.20%) on studied attributes of mint in Pb contaminated soil.

**Figure 9:**
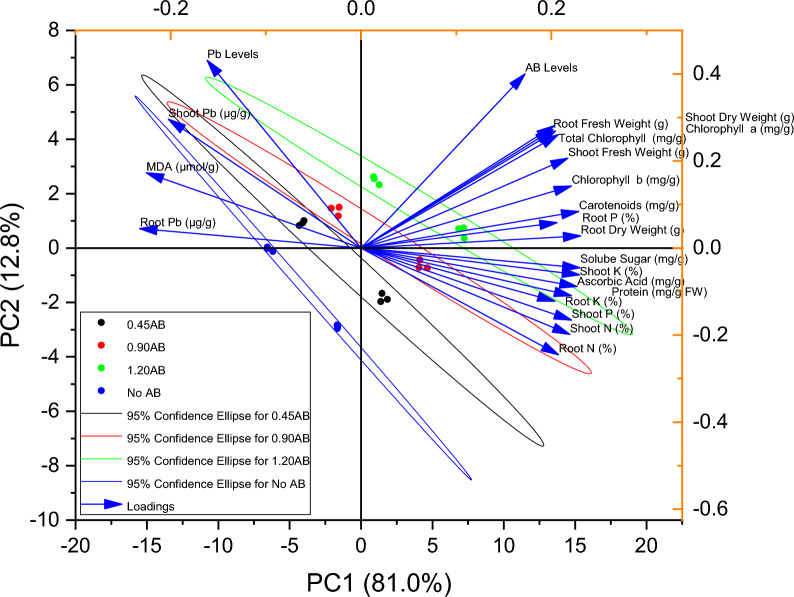
Principal component analysis for various levels of acidified biochar (0%, 0.45%, 0.90%, 1.20%) on studied attributes of mint in Pb contaminated soil.

Pearson correlation showed that Pb levels showed significant negative correlation with root dry weight, ascorbic acid, soluble sugar, protein, chlorophyll b, carotenoids, shoot and root N, P and K. However, Pb levels were significant positive in correlation with MDA, shoot Pb and root Pb concentration in mint plants (Fig. 8). Principal component analysis showed that root Pb, shoot Pb and MDA were more closely associated with the applied Pb levels. Root and shoot fresh weight, chlorophyll contents, root and shoot N, P and K were more closely associated with AB levels. Majority of improvement in growth attributes, chlorophyll contents and nutrients were more under the influence of 0.90 and 1.20AB (Fig. 9).

**Figure 10:**
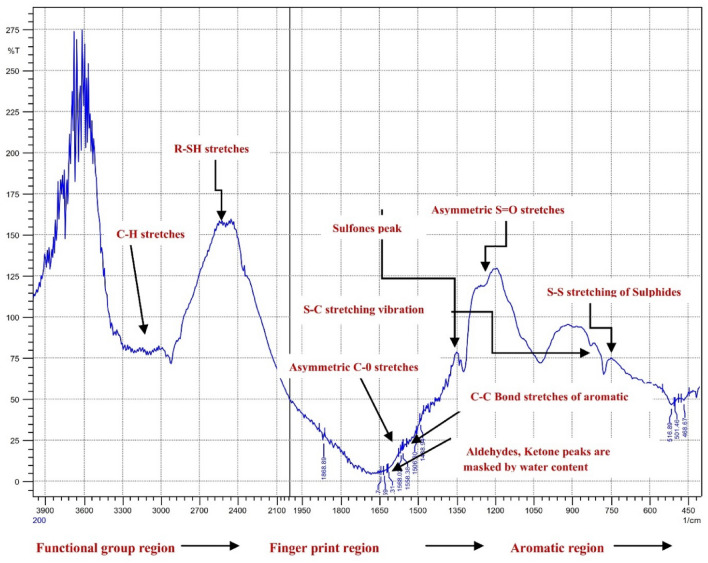
FTIR spectra of Acidified Biochar in functional group, fingerprint and aromatic region.

The bond vibrations, including stretching and bending of functional groups, as well as the fingerprint region, were identified through peak-by-peak correlation using the FTIR spectroscopic data. Table 2 summarizes the results. A doublet was observed in the biochar spectra at 2330–2360 cm^−1^, which is attributed to a higher amount of CO_2_ compared to the atmospheric level, likely due to its adsorption within micro-pores. The biochar spectra showed weak responses for aromatic C–H and aromatic C=C at 885 cm^−1^ and 1575 cm^−1^, respectively, indicating the presence of only a small amount of aromatic bonds. The peak at 3167 cm^−1^ corresponds to the aromatic C–H stretching of lignin aromatic residues. However, it could also be the contribution of the aromatization of cellulose residues during pyrolysis. The strong peak at 3167 cm^−1^ in the spectra of long alkyl chains shows the presence of waxes in the biochar. The intensity of the –CH_2_– peaks at 3167 cm^−1^ can be used to estimate the amount of waxes present in the biochar. A broad, asymmetrical peak at ~ 2586 cm^−1^ corresponds to R-SH stretching in biochar, which is due to a diversity of R–SH containing functional groups such as thiols. The peaks near 1605 cm^−1^ can be assigned to C–C bond stretching resulting from aromatic rings of lignin, as well as newly carbonized and aromatized material from dehydration and cyclization of carbohydrate rings during pyrolysis. The FTIR spectra also showed peaks in the region of 1100–1300 cm^−1^, which could be assigned to S=O bond stretching in biochar. These peaks were numerous, overlapped, and difficult to assign in the averaged spectrum. Figures 10 show the spectra with the identified peaks.

**Table 2:** FTIR vibrational modes assigned to various functional groups in general biochar^32^.

Wave numbers (cm^−1^)	Functional groups
3600–3200	O–H
1630–1650	Carboxylate anion
1650–1780	Carbonyl/carboxyl functional group
2800–3000	C–H stretching band appears
3000–2840	Waxes and oils
3500–2800	Stretching of O–H and C–H give vibrational modes
1800–1500	Presence of functional group and carbonyl group due to interference of C–H bending
1650–1780	Carbonyl/carboxyl functional group due to strong band around
1630–1650	Carboxylate anion
~ 1600	Carboxylate anion frequency shift due to H– bonding
3200–3500	Multitude of O–H stretching frequencies in cellulose
2500–2600	Thiols correspondence to S–H stretching
600–800	Characteristic range of sulphides, S–H stretching vibration
1000–1300	Range of sulphoxides due to S=O stretching
1100–1300	Sulfones ranges

## Discussion

Lead (Pb) toxicity can significantly affect plants by inhibiting their growth and development^44^. When plants absorb lead from the soil, it accumulates in different plant parts, including the roots, stems, and leaves, causing various physiological and biochemical changes in the plant^45^. Lead toxicity in plants can have various adverse effects, one of which is the inhibition of photosynthesis. This is because lead can interfere with the plant's ability to absorb and utilize essential nutrients such as nitrogen and phosphorus, which are necessary for proper photosynthesis. As a result, the chlorophyll content and efficiency of the photosynthetic apparatus can be reduced, leading to decreased photosynthesis and growth in plants. Other effects of lead toxicity on plants include damage to the cell membrane, reduced water uptake, and impaired root growth^46^. The reduced photosynthetic activity caused by lead toxicity can lead to stunted growth and reduced biomass production due to the plant's reduced ability to produce energy. Additionally, lead toxicity can cause oxidative stress in plants by accumulating reactive oxygen species (ROS), which can damage the plant's cellular structure and affect its physiological functions. This can lead to a reduction in plant growth and development. Furthermore, lead toxicity can also affect the plant's water relations by causing water stress, which can further impair the plant's growth and development^47,48^.

Acidified carbon can promote soil microbes, improve root and shoot length, and increase fresh and dry weight in saline soils through several mechanisms. Firstly, acidified carbon can decrease alkaline soil pH by neutralizing acidic components in the soil, creating a more hospitable environment for microbial activity^49^. Biochar can potentially mitigate the negative effects of lead toxicity on plant growth by providing a range of benefits to the soil. For example, the application of biochar can enhance the population and activity of beneficial soil microbes, such as nitrogen-fixing bacteria, which can increase nutrient availability for plants^50^. This can improve the plant's ability to absorb essential nutrients such as nitrogen and phosphorus, which are crucial for photosynthesis, and thus help mitigate the negative effects of lead toxicity on photosynthetic activity and plant growth. In addition, the application of biochar can improve soil structure by increasing the organic matter content, which can improve the soil’s water and nutrient holding capacity^22^. This can help alleviate the effects of salt stress on plant growth and promote root development. The improved soil structure can also help to reduce the mobility of lead in the soil and prevent its uptake by plants^51^. Acidified carbon can release protons, which can bind to lead ions, forming complexes that are less mobile and less available for uptake by plant roots. Another mechanism is adsorption. Activated carbon has a large surface area and high porosity, which can adsorb lead ions and prevent them from interacting with soil particles or plant roots. The adsorption capacity of activated carbon depends on its surface chemistry and the concentration of lead in the soil solution^52^. Acidified carbon can also improve soil properties, such as pH and organic matter content, which can indirectly immobilize lead. For example, activated carbon can increase soil pH, reducing lead solubility and enhancing lead precipitation in soil^53,54^. Acidified carbon can also increase soil organic matter content, improving soil structure and reducing lead mobility by increasing its retention capacity.

Furthermore, better uptake of nitrogen (N), phosphorus (P), and potassium (K) in shoots and roots can decrease lead (Pb) toxicity in several ways^55^. Pb is a toxic heavy metal that can accumulate in plant tissues and interfere with various physiological processes, including nutrient uptake and utilization. When N, P, and K are taken up more efficiently by plant roots, it can reduce the uptake of Pb, as these nutrients compete with Pb for uptake sites in the roots. The uptake of N, P, and K can also improve plant growth, which can enhance the ability of plants to tolerate Pb stress^56^. Moreover, these nutrients play critical roles in plant metabolism, including the production of antioxidants, which can protect plant tissues from oxidative damage caused by Pb toxicity. For example, phosphorus is a crucial component of adenosine triphosphate (ATP), which provides energy for cellular processes. At the same time, nitrogen is an essential component of amino acids, the building blocks of proteins. Potassium involves various physiological processes, including water regulation and enzyme activation^57^.

## Conclusion

In conclusion, acidified biochar (AB) is an effective amendment for improving mint growth and nutrient concentration in saline lead-contaminated soil. All of the AB treatments improved the growth and biomass of mint, with the 1.20% AB treatment being the most effective. The application of AB also increased the nutrient content, including N, P, and K, as well as the protein and soluble sugar content of mint, while reducing the MDA content. Additionally, the application of AB decreased the uptake of Pb by the roots and shoots of mint, thereby reducing salt and lead toxicity in the plant. These findings suggest that the application of 1.20% AB amendment is a promising approach for mitigating the negative impact of saline and lead-contaminated soil on the growth of mint. However, further research is needed to validate these results under field conditions.

## Data Availability

All data generated or analysed during this study are included in this published article.
